# Barriers and facilitators of access to maternal, newborn and child health services during the first wave of COVID-19 pandemic in Nigeria: findings from a qualitative study

**DOI:** 10.1186/s12913-022-07996-2

**Published:** 2022-05-06

**Authors:** Godwin O Akaba, Osasuyi Dirisu, Kehinde S. Okunade, Eseoghene Adams, Jane Ohioghame, Obioma O. Obikeze, Emmanuel Izuka, Maryam Sulieman, Michael Edeh

**Affiliations:** 1 Department of Obstetrics and Gynaecology, College of Health Sciences, University of Abuja/University of Abuja Teaching Hospital, Gwagwalada, Nigeria; 2 Population Council, Utako District, 16 Mafemi Crescent, Abuja, Nigeria; 3 Department of Obstetrics and Gynaecology, College of Medicine, University of Lagos, Idi-Araba, Lagos, Nigeria; 4 Research Hub Africa, No 3, Atabara Street, off Cairo Street, Wuse II, Abuja, Nigeria; 5 Lifesworth- Research Lab II, Abuja, Nigeria; 6 Department of Community Medicine/Public Health, Federal Medical Centre, Bayelsa, Nigeria; 7 Department of Obstetrics and Gynaecology, University of Nigeria, Nssuka, Enugu, Nigeria; 8 Department of Obstetrics and Gynaecology, Muhammad Abdullahi Wase Teaching Hospital, Kano, Nigeria; 9 Department of Obstetrics and Gynaecology, General Hospital, Taraba State, Takum, Nigeria

**Keywords:** COVID-19, Maternal, Newborn, Child health, Services, Access, Nigeria

## Abstract

**Background:**

COVID-19 pandemic may have affected the utilization of maternal and newborn child health services in Nigeria but the extent, directions, contextual factors at all the levels of healthcare service delivery in Nigeria is yet to be fully explored.

The objective of the study was to explore the barriers and facilitators of access to MNCH services during the first wave of COVID-19 pandemic in Nigeria.

**Methods:**

A qualitative study was conducted among different stakeholder groups in 18 public health facilities in Nigeria between May and July,2020. In-depth interviews were conducted among 54 study participants (service users, service providers and policymakers) selected from across the three tiers of public health service delivery system in Nigeria (primary health centers, secondary health centers and tertiary health centers). Coding of the qualitative data and identification of themes from the transcripts were carried out and thematic approach was used for data analyses.

**Results:**

Barriers to accessing MNCH services during the first wave of COVID-19-pandemic in Nigeria include fear of contracting COVID-19 infection at health facilities, transportation difficulties, stigmatization of sick persons, lack of personal protective equipment (PPE) /medical commodities, long waiting times at hospitals, shortage of manpower, lack of preparedness by health workers, and prioritization of essential services*.*

Enablers to access include the COVID-19 non-pharmacological measures instituted at the health facilities, community sensitization on healthcare access during the pandemic, and alternative strategies for administering immunization service at the clinics.

**Conclusion:**

Access to MNCH services were negatively affected by lockdown during the first wave of COVID-19 pandemic in Nigeria particularly due to challenges resulting from restrictions in movements which affected patients/healthcare providers ability to reach the hospitals as well as patients’ ability to pay for health care services. Additionally, there was fear of contracting COVID-19 infection at health facilities and the health systems inability to provide enabling conditions for sustained utilization of MNCH services. There is need for government to institute alternative measures to halt the spread of diseases instead of lockdowns so as to ensure unhindered access to MNCH services during future pandemics. This may include immediate sensitization of the general public on modes of transmission of any emergent infectious disease as well as training of health workers on emergency preparedness and alternative service delivery models.

**Supplementary Information:**

The online version contains supplementary material available at 10.1186/s12913-022-07996-2.

## Background

Corona Virus-19(COVID-19) was declared a public health emergency of international concern by World Health Organization on Jan 30,2020 concern [1]. Nigeria’s index case was confirmed on 27^th^ February 2020 and the number has steadily risen to 161,074 with 2,018 deaths as of 16^th^ March 2021 [2].

Nigeria alone with a maternal mortality ratio of 917 per 100,000 live births contributed up to 23% of global maternal deaths in 2017 [3]. Newborn and child health indices in Nigeria are also amongst the worst in the world [4] with neonatal mortality and under mortality rates of 32.9 deaths per 1000 live births and 100.2 deaths per 1000 live births in 2017, respectively [5]. The poor maternal and newborn child health (MNCH) indices have been attributed to several factors including poor political will, weak health systems and lack of access to MNCH services [6]. For example, only 4 in 10 births are delivered in health facilities while just 43% of births are assisted by a skilled provider [7].

Experiences from the Ebola Epidemic in West Africa showed that access to MNCH services is likely to be disrupted during public health crisis [8]. With advent of COVID-19 pandemic, the resilience of health systems, their levels of emergency preparedness and the response of nations were tested and found to be fragile in most instances [9]. This portends great danger for a country like Nigeria where access and utilization of maternal, newborn and child health services are still poor [7].

During the first wave of the COVID-19 pandemic, Nigeria instituted a series of stringent non-pharmaceutical interventions, including several phases of stay-at-home orders and cessation of non-essential movements and activities (lockdowns) from 30^th^ March, 2020 to 15^th^ July, 2020 [10]. These measures were aimed at curtailing the spread of the virus while giving the government opportunity and time to put in place other measures like establishment of testing centers and procurement of PPEs. Lagos state in addition to the above measures increased the hazard allowance of health workers from US$13 to US$65. This was aimed at increasing the morale of the health workers [11].

COVID-19 pandemic may have affected the utilization of maternal and newborn child health services in Nigeria but the extent, directions, contextual factors at all the levels of health care service delivery as well as perceptions of patients, health workers and policymakers regarding these changes have not been evaluated in the same study in Nigeria. Additionally, the side effects of lockdown and movement restrictions instituted by the government to reduce community spread on maternal health in Nigeria have so far been largely unexplored.

The objective of the study was to explore the barriers and facilitators of access to MNCH services during the first wave of COVID-19 pandemic in Nigeria.

## Methods

### Study design

The study utilized a qualitative study design to explore the perceptions of users of healthcare facilities, health workers, and policymakers on how COVID-19 has shaped the utilization of MNCH services as well as other contextual factors contributing to the projected views across six states of Nigeria.

### Study settings

The states were chosen purposefully to represent the six (6) geopolitical zones of the country. Three states namely Abuja, Lagos and Kano had high cases for COVID-19 while the other three (Enugu, Taraba and Bayelsa) had fewer cases of Covid-19. Three Local Government Areas (LGA) were selected from each state representing three senatorial districts. The selection of states with high and few cases was considered necessary to explore contextual differences in barriers and facilitators to accessing MNCH services in these states. The states, health facilities and the number of covid-19 cases in the states as of 17^th^ May 2020 when data collection began is as shown in Table 1.

**Table 1:** List of participating states, health facilities and number of cases of COVID-19 in the states

Level of health facility	**High COVID-19 burden states and number of cases of COVID-19 in the states as of 17**^**th**^** May, 2020**			**Low COVID-19 burden states and number of cases of COVID-19 in the states as of 17**^**th**^** May, 2020**		
	***Abuja FCT*** 397	***Lagos*** 2,373	***Kano*** 844	***Taraba*** 17	***Enugu*** 11	***Bayelsa*** *6*
Tertiary health centre	University of Abuja Teaching Hospital, Gwagwalada, Abuja	Lagos University Teaching Hospital, Idi-Araba, Lagos	Muhammad Abdullahi Wase Teaching Hospital, Kano	Federal Medical Center Jalingo, Taraba	University of Nigeria Teaching Hospital, Ituku-Ozalla, Enugu	Federal Medical Center, Yenagoa
Secondary health centre	General Hospital, Kwali	General Hospital, Badagry	Wudil General Hospital, Wudil	General Hospital, Sunkani Ardokola	Poly General Hospital, Enugu, North LGA	Diete-Koki Memorial Hospital, Opolo
Primary health centre	Comprehensive Health Center, Abaji	Comprehensive Health Center, Isolo	Comprehensive Health Center, Kiru	Rifkatu Danjuma maternity PHC, Takum	Comprehensive Health Center Obukpa, Nsukka	Amarata Primary Health Care Center

### Study participants and data collection procedures

A total of 54 in-depth interviews (IDIs) were conducted across all six (6) states with 9 interviews in each state comprising of 3 policymakers, 3 service providers and 3 service users. These were spread equally across the three levels of health care systems (Primary health care, secondary health care and tertiary healthcare) in the states. The state study coordinators scheduled and confirmed the dates and time of the planned IDIs with the study participants after obtaining informed written consent. The participants were also informed that the interview will be recorded during the informed consent process. Interviews were facilitated by experienced interviewers over the phone based on prior schedules by study coordinators. All interviews were conducted with study participants using their personal phones, although this was not a criterion in the selection process. The interviewers (three female and three male) were experienced qualitative researchers with extensive training and expertise conducting research across Nigeria. They worked with members of the core research team to schedule interviews with the respondents while determining the best time for the interview to take place. The study participants were informed about the purpose of the study and were invited to participate in the interview, which lasted for approximately 20 to 30 min. All interviews were conducted in English language using an IDI guide designed specifically for this study for each of the stakeholder groups (Maternal and child health service users, service providers and policy makers across all levels of healthcare system). In-depth interviews (IDI) guides captured barriers and facilitators that influenced access to MNCH services and service delivery during COVID-19. The final research tool was tested amongst each stake holder group before utilization for the study (See Supplementary file 1).

### Conceptual framework (the three delays model)

The conceptual framework for understanding the impact of COVID-19 on MNCH service utilization in this study was the three delays model. The delay model was used to explore delays in access to MNCH services in three different but closely related phases [12–14].

### Three (3) delays model

- Phase I Delay: Delay in deciding to seek care: These are individual or familial factors such as socio-cultural, economic factors, illness characteristics and perceived quality of care.
- Phase II Delay: Delay in reaching healthcare facility: These include distance to health facilities, transport cost, availability of transportation and poor road networks.
- Phase III Delay: Delay in receiving adequate care at the health facility: These include waiting/response time at the facility, shortage of supplies/equipment, the competence of available personnel, adequacy of the referral system and quality of care.

### Data analysis

The interviews were recorded digitally, transcribed verbatim, and transferred to NVivo12 software for analysis. The codebook development process entailed a review of all the transcripts by four researchers (OD, ES, JO GOA) who contributed to the development of a thematic framework of codes through consensus. Thematic analysis was used as an analytical strategy to explore patterns and themes within the data. Thematic analysis involves the identification, analysing and reporting of patterns in data and provides the basis for many other forms of qualitative analysis [15]. The process of thematic analysis involves careful identification of themes achieved through familiarization and immersion in data [16]. The steps involved in the analysis process include familiarization with the data; initial coding and development of a codebook; search for themes by reviewing, recoding and categorization of data; review of themes; and definition of final themes [15]. A deductive analytical approach was used in this study because the general aim of thematic analysis was to test a previous theory in a different situation [17, 18]. Some codes were determined as priori codes and others emerged during the coding process. As part of the coding process, the research team explored the data until data saturation was achieved when additional interviews coded did not change the structure/content of the codebook. The process of identifying themes highlighted contextual situations that underpin perceptions and experiences expressed in the data.

The themes were organized using the three (3) delay models to explore the contextual factors that shaped utilization as well as enablers and barriers of access.

## Results

Demographic characteristics of study participants are presented in Table 2. The results are presented under two major sections: Barriers and facilitators of access to MNCH services. The barriers of access to MNCH services are broken down into three domains: delay in seeking care, delay in reaching care and delay in receiving care. On the other hand, facilitators of access to MNCH services were limited to two domains (decision to seek care and receiving care at the hospitals). Direct quotes from the interviews were used to illustrate the results at each level of delay or facilitation. Majority of the services users were aged below 40 years reflecting the age category that utilizes MNCH services. Majority of the service providers were female, and nurses/doctors. Majority of policymakers were male (Table 2).

**Table 2:** Participant’s demographics

DESCRIPTION		Number of interviewees (*N* = 54)
Service Users (SU)		18
Service Providers (SP)		18
Policy Makers (PM)		18
Demographic features for service users		Number of SUs (*N* = 18)
Age Group	20 – 30	7
	31 – 40	10
	41 – 50	1
Demographic features for service providers		Number of SPs (*N* = 18)
Age Group	20 – 30	1
	31 – 40	5
	41 – 50	5
	51 – 60	7
Category	CHEW	1
	Matron/ Nursing Officers	7
	Nurse-in-Charge	4
	Resident Doctor/ Medical Officer	3
	Consultants OBS/GYN	3
Gender	Male	4
	Female	14
Demographic features for policymakers		Number of PMs (*N* = 18)
Age Group	30 – 40	4
	41 – 50	11
	51 – 60	3
Gender	Male	13
	Female	5

The summary of major themes is shown in Table 3.

**Table 3:** Summary of findings

Determinants of utilization of MNCH services using the Three Delays Mode	
**Barriers to access to MNCH services**	
*Delay in seeking care*	•*Socio-economic factors* •*Fear of contracting COVID-19 at health facilities*
*Delay in reaching health care facility*	•*Lack of transportation* •*Movement restriction during the lockdown and harassment by security agents*
*Delay in receiving care at the hospital*	•*Long waiting times and a daily capped number* •*Patient’s non-compliance with the” no- facemask, no entry” rules* •*Inadequate PPEs* •*Stigmatization of service users by health workers* •*Shortage of manpower, lack of incentives and prioritization of essential services*
**Facilitators of access to MNCH services**	
*Enablers of decision to seek care*	Community sensitization on healthcare access during the pandemic
*Enablers of access to receiving care at the hospital*	•*COVID-19 non-pharmacological measures instituted at the health facilities* •*Adaptive strategies to reduce waiting time at health facilities* •*Adaptation of service delivery structure and COVID-19 safety protocols* •*Training and supportive supervision for health workers* •*Increment in hazard allowance to Health workers*

### Barriers to access to MNCH services

#### Delay in seeking care

Several factors influenced the decision making of women to visit health facilities and access MNCH services during the COVID-19 pandemic.

##### Socio-economic factors

Decision making around care-seeking was influenced by socio-economic drivers of utilization. Petty trading was the means of livelihood of women in most communities; during the nationwide COVID -19 lockdown, majority of these women were unable to sell their products and did not have disposable funds to pay for MNCH services. The consequence of this was the inability of women to afford MNCH services at facilities. The increased cost of transportation during the lockdown period and the associated cost of procuring personal protective equipment (PPEs) like face masks required to visit clinics constrained the capacity of users to access care.

R: because they are poor, even the face mask some of them couldn’t afford the face mask because for days they’ve not gone out they couldn’t go to the market, there was no sale, there was no movement even the transport was very expensive so that’s really a very important factor - **Enugu _Policy Maker _Primary Health Centre**

R…if I no go market, how can I get money to buy food for my children, how can I get money to go that antenatal – **Taraba_ Service User_ Secondary Health Centre**

R: You know our people are not rich. Some people go to the market sell and eat the same day…so this really has affected them and if you don’t have money to eat you won’t have money to go to the hospital and some of these hospitals you know are not cheap… You understand…**Bayelsa _Service Provider__ Secondary Health Centre**

##### Fear of contracting COVID-19 at health facilities

The fear of contracting COVID-19 at the facilities were key barriers to access to MNCH services during the lockdown. Health facilities were viewed as high-risk centers for contracting COVID-19 as it was widely publicized that health workers were contracting the virus. Service users were also concerned about the availability of service providers to attend to them at the facilities as there was limited information about the availability of health services during the lockdown. Religious beliefs that the COVID-19 situation required divine intervention was also a barrier that kept potential service users from accessing health facilities during this period.

R: So there are some group of patients that voluntarily told us that "I stopped coming to your hospital because I am afraid of this virus” …some because of the transportation, and some this financial issue because they complained that during this lockdown, you know the economy, how it has gone down so their husbands cannot provide some financial support to them. Some patients told us that it is due to fear of COVID-19. – **Kano_ Service Provider _Tertiary Health Centre**

R: Yes, I was anxious when he just started little fever. How can I even be able to access the health facility because how will I even go to the health facility? What am I going to do there? Would there be health personnel to attend to me? And when I get there, won't I be infected? - **Abuja _Service User_ Tertiary Health Centre**

R: … People are afraid now to go to the hospital… Because of fear of being infected by the Covid… - **Bayelsa _Policy Maker _Secondary Health Centre**

#### Delay in reaching care

##### Transportation difficulties

Service users experienced significant delays in reaching health facilities due to transportation difficulties. The number of transportation service providers significantly reduced during the lockdown. Potential service users who had their vehicles found it easier to visit the hospitals than those who were dependent on public transportation. Public transportation became more expensive and difficult to access especially in remote or rural areas and some people had to resort to walking to facilities to access care.

R: Especially at this period, there are times that even some of the patients will come from far places, you’ll see them looking so fagged out. Last week, I encountered a case. I say aah 'what's the problem?' She said 'I couldn't access any transportation to come.**' - Abuja _Service Provider _Secondary Health Centre**

R: The delay in getting to hospital that's the major problem. That was the major problem then, before the ambulance could go round to pick people. Actually, that was the major problem during the lockdown, accessibility to the hospital. We the workers couldn’t get to the hospital on time *–* Lagos_ Service Provider_ Secondary Health Centre

R: Transportation for example, if you want to get to a health facility, you need to be able to move, means of transportation, everybody was locked down so there is no how you can easily get to health facilities, if you don’t have it in the house, the public transportation system are down during the time of the lock-down, so people may have to necessarily maybe trek to the nearest health facilities to attend it or be able to... if you have means of moving, personal means of transportation to be able to get to where you can acquire those services, those were the challenges I was talking about. **Taraba Policy _Maker _Tertiary Health Centre**

##### Movement restriction during the lockdown and harassment by security agents

During the lockdown, in addition to the restriction of movement, there was a curfew in place. Security agents were positioned on the road at checkpoints to enforce the lockdown. There was a consensus among service users and providers that these checkpoints created delays in reaching the hospitals. There were reported cases of security agencies harassing commuters and requesting for proof that they were going to the hospital in some instances. The experience with security agents varied as some service users, mentioned that although there were delays at checkpoints, they could continue their journey after they presented their hospital identity cards or medication they were previously given at the hospital.

R: Yeah, there are some delays because of the check points. The check points, you know are very plenty on the roads, like I was watching it on television when I saw a woman saying she came to the hospital and had to go home and bring her things back, but the person has been stopped miles away from the check point and they are checking them one by one, so if this kind woman is in labor, she will deliver on the way or something may happen. So, some of them have delay in getting to the facility because of the restriction in movement. And there is nothing they can do about it especially if it is not your own transport. **Abuja_ Service Provider_ Tertiary Health Centre**

R: The lockdown affected everybody. Once you have your facemask and you tell them (security agents) you’re heading to the hospital, and you have any evidence as in any card to show them they will believe it. Then they’ll allow you to pass but apart from that, if you just tell them you’re heading to hospital, they will not allow you but once you insist, they’ll rather take you there by themselves. Yes, the last time I was sick, the last time, that was exactly what they did to us, I was telling them that my baby was sick (laughter). Yes, but my baby was not feeling fine, they refused because that was my first time. They had to take us there by themselves and I said it’s even better it’s like they know I don’t have enough money to pay the bike man. They had to take me there by themselves and we see the doctor and we left, so while coming back still that the same road. I had to show them the medicine collected there so that was how they allowed me now. – **Taraba_ Service User_ Secondary Health Centre**

#### Delay in receiving care

##### Long waiting times and a daily capped number of patients to be attended to at the hospitals

Due to the measures that were instituted to keep service users and providers safe at the hospitals, waiting time at health facilities increased. In addition, some facilities reduced the number of health personnel attending to patients and this resulted in further delays in receiving care. Health facilities capped the number of services users receiving care daily and when the number of patients seeking care exceeded the allotted number, they were turned back and asked to return on a later date. This implied that service users had to visit the facilities very early to be included in the list of patients to be seen each day; this increased waiting time and some service users waited and did not get attended to. Additionally, this was said to have resulted in poor quality of care received by service users.

R: Well, the thing is that they are many, they attend to you quick but now they are not attending to us quickly as it used to be. You will stay longer before you go home. You will go and buy your drugs and you will go, but now you will stay longer before they attend to you because they are not giving us the care again! There are just two or three personnel that you will see to attend to the large number of women. – **Enugu_ Service User _Primary Health Centre**

R: …You get to the hospital around 7am, you’d have to leave around 12pm. I think the doctors there are not enough, we’d have to sit and sit and sit, you know. It’s time consuming. That’s the only challenge I have oh! if you gather as early as 7:30am, 6:30am but you’d have to stay till 12:00pm sometimes, or 2:00pm which is very frustrating. – **Bayelsa_ Service User_ Primary health Centre**

R: we wanted to stick to twenty patients per day, because of social distancing and we wanted to avoid overcrowding. So, we only attend to twenty clients daily, yes, twenty per day. we run our services Monday to Fridays, so we ask them to come in their twenties so most of the time we have more than twenty and we have to turn back others, so it’s been a problem. -**Lagos _Service Provider _Primary Health Centre**

##### Patients’ non-compliance with the “no- facemask, no entry” rules

Some service providers reported that when patients presented to the health facilities without facemasks, they were declined treatment, and this caused significant delays in receiving care in instances where these patients could not afford to buy facemasks. Service providers felt vulnerable and at risk of contracting COVID-19 if they provided services to patients who did not wear masks. The hospitals were unable to provide face masks for patients who were made to buy them at very high costs from hawkers at the hospitals. Service users corroborated this and reported that they experienced significant delays due to non-use of face masks. Some reasons given by service users for not wearing facemasks was they could not afford to buy facemasks, and they felt a chocking sensation when they wore the masks.

R: …initially when you ask them to use face mask, they will be telling you its chocking them, we don’t have the money to buy. - **Kano _Policy Maker _Secondary Health Centre**

R:Two weeks ago there was a patient that had ectopic (pregnancy), she collapsed while in the market so it was with the intervention of the COVID-19 committee in the hospital that she was brought in as they made a made case for her that this is not a COVID-19 but a gynae emergency…The instruction from the state is that no face mask no entry, so it has really been difficult to really assess patients that need our services once they cannot afford the hundred naira face mask, they turn them out, they can’t enter the hospital. **Lagos_ Policy Maker_ Secondary Health Centre**

##### Inadequate PPEs and Medical Commodities

There was a consensus among policymakers and service providers that the inadequacy of PPEs affected the capacity of health facilities to respond effectively to the pandemic and maintain optimal service delivery levels. Some service providers had to purchase PPEs with personal funds to ensure they were safe and could continue delivering health services. In other instances, patients were asked to buy PPEs as part of the service delivery process. Policymakers, especially those at primary healthcare facilities, reported that PPEs were only procured for health facilities at the initial phase of the pandemic. Subsequently, the government was unresponsive to their pleas to provide more PPEs. The only PPEs that were made available were facemasks and hand gloves; facilities were not provided with face shields, gowns, and boots. The lack of information or protocols to guide the response of healthcare workers to the COVID-19 pandemic and safely continue service delivery made it more difficult for service providers.

R: Yes, the challenges we faced is that at the initial stage when the adequate PPEs were not provided to my staff, the other colleagues and my department, we actually faced challenges of how we can protect ourselves and other patients and our families from this disease, COVID-19, especially when we have a suspected case and when we get this virus, how can someone be taken care of by the health system... – **Kano_ Service Provider_ Tertiary Health Centre**

R: The biggest challenge was the absence of personal protective equipment, and lack of information because the facility was not given direct, first-hand information, and so we were... scouting for information by ourselves on the web, on the internet, looking for information for yourself, there was no action plan, there was no protocol, everyone was just you know scrambling to do things for yourself as you can, there was obviously no personal protective equipment provided, so people had to use their monies… to buy these things for themselves, to protect themselves, and there was just total lack of leadership – **Lagos_ Service Provider _Tertiary Health Centre**

R: So over here... I have never...heard of personal protective equipment; I see them on the TV but no... not even one has been provided for me here. Except about two months ago, they gave us about 100ml of sanitizer… I bought a packet of mask, at the cost of fourteen thousand naira. – **Enugu_ Service Provider _Primary Health Centre**

##### Stigmatization of service users by health workers

Service users were stigmatized by health workers once the patients have any symptom that may be associated with COVID-19 even when they do not have the disease. The lack of testing capacity in the hospitals worsened these problems as patients were refused care in the hospitals until they presented COVID-19 test results.

R: I know of a woman who did not receive attention from the health workers because she had cough and difficulty in breathing. They insisted that she must present a COVID-19 test result. At the end of the day, it was a case of cardiac failure and she nearly died. **Abuja _Service User_ Tertiary**

R: Before now, in fact everything was like 0%, people are afraid to come to the health facility. Health workers would not want to be in their primary place of assignment for fear of Covid 19, somebody will present with malaria symptoms, 'eeh this is COVID-19 oo' the next thing will be for them to run to their houses, Patients with symptoms are stigmatized. But we have to continue to educate them that no, it’s not every person you see on the street or any symptoms that are similar to Covid-19 that is Covid 19… - **Bayelsa _Policy Maker _Primary Health Centre**

##### Shortage of manpower, lack of incentives and prioritization of essential services

Majority of the tertiary facilities experienced a significant shortage of health personnel because these staff also doubled as health care providers at the various isolation centres. Health managers in some facilities were instructed to reduce the number of health personnel involved in the response during the lockdown and this resulted in an increased workload for health workers responding to the healthcare crisis. This increased workload resulted in delays in the delivery of MNCH services. No additional incentives were provided for the healthcare workers who had to run extra shifts during the pandemic. This significantly affected the staff motivation and impacted on welfare of health workers who were afraid to return home in between shifts to avoid infecting their families. A policymaker reported that health workers would have been better motivated if incentives were provided to support on-site accommodation and a daily allowance during the lockdown.

R: …these healthcare workers have to be there every day with no form of motivation or anything. Their feeding, accommodation is a problem because you have to think of the worries if they go back home to their families, won’t they be infecting them? **Bayelsa_ Policy Maker _Tertiary Health Centre**

R: That time they declared lockdown, an order came from our parent hospital, that we should cut down manpower either to sixty percent... which we did…That means that if every morning, we were having up to ten nurses on morning duty… during the pandemic we reduced to five. - **Enugu _Service Provider_ Primary Health Centre**

R: That one is something that we have been battling with… but even before COVID you know that man power is never enough especially in a setting like this because the work load here is very tasking because we have many patients. What we see is that other centers refer their patients here, so we work... work is actually tasking here, and staff are never enough, you know but we manage. **Abuja_ Service Provider_ Tertiary Health Centre**

### Facilitators of access to MNCH services

#### Facilitators of decision to seek care

##### Community sensitization on healthcare access during the pandemic

Factors that positively influenced utilization of MNCH services as reported by policy makers include community-level COVID-19 sensitization. Some of these sensitization meetings involved community and religious leaders to improve service utilization among community members. In addition, febrile illnesses such as malaria which could be easily treated were perceived as COVID-19; the sensitization sessions enlightened community members about the different scenarios and the need to seek appropriate care. Community sensitization was also useful in communicating to patients on the need to wear face masks as well as cost-effective ways of obtaining and using facemasks.

: Okay at our setting, like in the primary health care, immediately we ... by early April, we had already called for a meeting at the FCT public health department and agreed that we should start community sensitization amongst the religious leaders, policy makers and traditional leaders, - **Abuja_ Policy Maker_ Primary Health Centre**

R: Yes, access to services is really a problem, with the significant advocacy, consistent engagement with our stake holders, the front-line health care workers, I think we have been able to see a little improvement in utilization of services. - **Bayelsa _Policy Maker_ Primary Health Centre**

R: Actually, the level of adherence is steady, because we see them when they go, when they are coming the next time, we see them what you tell them they are adhering to, initially when you ask them to use face mask, they will be telling you its chocking them, we don’t have the money to buy, but when you explain to them these are the rational on how you use this face mask, some of them they even use plain clothes and sew it to wear it to cover their nose and mouth, it also helps them, by educating them, they actually adhere to it. - **Kano _Policy Maker _Secondary Health Centre**

### Facilitators of access to care at the hospitals

#### COVID-19 non-pharmacological measures instituted at the health facilities

Some service users opined that the good hygiene practices and precautionary measures observed at health facilities encouraged them to access care and allayed their fears about contracting COVID-19 at the facilities. In addition, the health education they received at the facilities was useful in clarifying misconceptions they had about COVID-19 transmission and facilitating access to MNCH services.

R: So, wherever we go before they even check your BP, they would sanitize the BP equipment’s. When you enter inside their clinics you will wash your hands and they provide you with sanitizers to sanitize your hands. They provide all these for the patients. **– Bayelsa _Service User_ Tertiary Health Centre**

R: Initially when we had not been lectured on how the thing spread, we thought if you come outside immediately you will be contaminated, so that is why we stayed in the house. Even the time we were supposed to go and register for ANC, we thought that the hospitals would not be working, but when we reached there, they opened our eyes, they lectured us on some certain things, so the fear now... the fear we had initially was no longer there now. - **Enugu _Service User_ Secondary Health Centre**

#### Adaptive strategies to reduce waiting time at health facilities

Some health facilities adapted to the challenges with service delivery during the pandemic by staggering ANC appointments and scheduling patients’ attendance to the hospital for treatment. Patients were encouraged to visit these facilities because they knew the health workers were committed to ensuring that they received care.

R: It is not somehow difficult because they won't waste our time. If we just come, those of us who will not be attended to are requested to comeback on a later date while they will ask others to wait. So, it is not difficult, it is easy. – **Abuja_ Service User_ Secondary Health Centre**

R: Right now, just as I told you, in the health talk, we use to tell them about personal and environmental hygiene and even err... their antenatal days, we are trying to shift it. No longer the regular schedules we were using. For instance, we can tell somebody to come in three weeks when ordinarily she is supposed to come in two weeks, you understand, so we now provide longer intervals between visits. For instance, come `in one month, come in two month and that is the directive of the government. They say we should be telling them like that because if they come and they become crowded, you will not be able to provide the covid-19 safety measures. So, we now gap their visit so that they don't come very close as we use to give them before. – **Enugu_ Service Provider_ Secondary Health Centre**

#### Adaptation of service delivery structure and COVID-19 safety protocols

Majority of policy makers and service providers reported that the service delivery structure was reorganized to be responsive to the demands of the lockdown period. In some facilities, MNCH care which was previously delivered to service users using a batched system on specific days was adapted to occur on a rolling basis. This implied that MNCH service users who visited these facilities for ANC and immunization services were attended to immediately they arrived at the facility. This was done to reduce large gatherings of service users at the facilities and improve social distancing. Health facilities also reduced the number of caregivers who accompanied MNCH service users to the facilities to limit large gatherings. Some facilities also instituted protocols for ANC visits, home visits for postnatal care and delivery. The protocols included guidance on the use of PPEs (face mask, gloves), temperature checks as well as ensuring the service provider and service user compliance to safety measures. Temperature checks were carried out at the gates before any service user or service provider was granted access to the health facility as well as handwashing and use of hand sanitizers. A policymaker mentioned that there were special arrangements for pregnant women who tested positive to Covid-19.

R: Any patient on admission has only one patient relative to stay with him and the nurse in charge has to know who the person is. You don’t just keep changing people for us. **Abuja _Policy Maker_ Secondary Health Centre**

R: Even those that delivered during this period, after 6 hours of post-delivery, we watch them and if they’re stable, we discharge them home, because the more they stay in the hospital, visitors will tend to come and visit them. -**Taraba_ Service Provider_ Primary Health Centre**

R: So essentially, all these things are present it’s just that the place is not as busy because we decreased hospital visits because for women for instance there’s a time of their gestation that they’re meant to come every two weeks…we increased some of them to four weeks… until a particular time. Those who are meant to be coming every week, we increased it to two weeks if they’re low risk; meaning low risk means they don’t have problems that we think that can get complicated you know, so we give such women a longer appointment so it makes the clinic lighter; so that the social and physical distancing can be practiced in clinic… So you know if you give them that long appointment time it will improve our physical distancing, so that makes the clinic a bit lighter. -**Bayelsa_ Service Provider_ Tertiary Health Centre**

#### Training and supportive supervision for health workers

Training and supportive supervision were provided for health workers and community volunteers on COVID-19 protocols, the preparedness of the health workers, use of PPEs and managing of suspected cases. Training of trainers’ sessions not only focused on COVID-19 but also on the care of women and children at the primary health care centers.

R: We had training of trainers… for health workers at the primary health care level in the communities…Society of Gynaecology and Obstetrics of Nigeria (SOGON) was represented, so many other organizations that are in the care for women and children were also represented at the meeting…we trained our health workers just for four days on COVID-19 response, preparedness of the health workers towards the COVID-19 pandemic. So, we taught them how to use PPE, the ones we must use always and the ones we use for a suspected case. We also had training of community volunteers. So that they will go to the communities… because you know rumors are causing panic amongst the community members which is now preventing them from accessing appropriate care or even notifying appropriately when we have suspected cases in the community… we finished the training last week but there will be ongoing supportive supervision. – **Abuja_ Policy_ Maker _Primary Health Centre**

R: okay, we have an infection prevention control at the health facility so prior to that we have had series of trainings so we had two trainings on infection prevention control and we decided to paste and give some IEC materials on infection which were displayed at different places in the facility, so the PPEs like I said earlier on at the beginning it was regular but towards the end erm is not end yet we are still in it and it sometimes there is always paucity of supply, it can about six weeks to get a new supply – **Lagos_ Policy Maker_ Primary Health Centre**

R: We have trainings, and all of us, all the health workers here including those that are cleaners, we have been retrained and they know what to do about it, they know about the hand hygiene, respiratory hygiene, the face mask and social distance, even the way we see patients is not as before where people have to seat anyhow…we have nurses, and some community health extension workers that work under the MNCH, they are trained specialist to even carry out their activities even with this issue of the COVID. – **Taraba_ Policy Maker_ Secondary Health Centre**

### Increment in hazard allowance to health workers

The Lagos state government in her bid to motivate and increase the morale of health workers in the state announced an increase in the hazard allowance of her workers from N5,000 to N25,000. This helped in making health workers who were reluctant in going to work to reconsider their stand thereby helping to address the problem of lack of health workers in some facilities.

R: At least the government has increased their hazard allowance. This has made some health workers to reconsider the situation and get back to work. At least they have gotten something from the agitations and COVID-19 has spurred the government to action -**Lagos_ Service User_ Secondary Health Centre**

R:. -We are happy about the increment in the hazard allowance and pray that every worker will benefit. It will really encourage health workers. **Lagos_ Service Provider_ Primary Health Centre**

## Discussion

This paper reports the perceptions and experiences of key stakeholders across the three levels of health systems in Nigeria on barriers and facilitators associated with access to MNCH services during the first six months of COVID-19 pandemic. It also explored other contextual issues that have shaped service delivery along the MNCH continuum of care during the COVID-19 pandemic.

Barriers to accessing MNCH services by patients in this study included fear of contracting COVID-19 at health facilities, lack of funds to pay for services at the health facilities, transportation difficulties, shortage of manpower, long waiting times and a daily capped number of patients to be attended to at the hospitals, negative attitude of healthcare workers, harassment by security agents, and stigmatization of service users by health workers.

A previous study that assessed the psychological impact of covid-19 amongst pregnant women in Italy had reported women’s concerns and anxiety regarding possibility of transmission of the disease to their babies [19]. This is also consistent with reports from a national survey in the United Kingdom [20] and another study from India [21] in which pregnant women were unwilling to seek maternity care at the hospital due to fear of the risk of coronavirus transmission to them or their babies. In a study by Davis et al., [22] it was found that women in New York, USA are preferring home deliveries instead of institutional deliveries due to the fear of being infected at the hospital. The delay in seeking care by pregnant women due to fear of getting infected with COVID-19 could lead to increased home deliveries with attendant complications.

Sustained health education of the public on the modes of transmission of any novel disease of public health importance as well as measures put in place at the hospitals to forestall transmission to patients would be useful in allaying fears, myths, and misconceptions. Unfortunately, the information presented to the Nigerian public by health workers on non-availability of personal protective equipment and the increasing number of deaths from COVID-18 amongst health workers at that time strengthened patients resolve to stay away from the hospitals.

Lockdown is an effective measure in slowing the spread of coronavirus around the globe [23] and was implemented in several countries including Nigeria to reduce community spread of COVID-19. Previous literatures have highlighted the challenges posed by the lock down especially as it relates to socio-economic losses [24]. Our study identified an indirect linkage between lock down and lack of access to MNCH services as patients could not afford to pay for health care costs or even transport costs to reach the hospitals due to halt of economic activities. A previous study on the impact of COVID-19 on health services utilization in Nepal also reported the negative implication of lockdown on access to MNCH services [25]. Another study from Sierra Leone, West Africa on the social consequences of COVID-19 showed that the lockdown had negative socio-economic impact on the population studied [26]. Drawing from these experiences, it may be wise to suggest the need for provision of social safety nets to cushion economic difficulties as well as allow free movement of pregnant women during any future lockdown.

Healthcare workers rely on personal protective equipment to protect themselves and their patients from being infected and infecting others. Our study showed that health workers in Nigeria lacked PPEs for the care and management of patients. This resulted in lack of confidence, low morale, and unwillingness of the health workers to provide services for fear of being exposed to COVID-19. Semaan and colleagues [27] had reported a similar perception by health workers. The World Health Organization in recognition of the universal shortage of PPEs during the period of this study had recommended its rationale use which involved using of PPEs based on the risk of exposure (e.g., type of activity) and the transmission dynamics of the pathogen (e.g., contact, droplet, or aerosol) [28].

A major problem that hampered health workers willingness to easily offer maternity services was the lack of incentives and health insurance policy for staff who felt that it was too risky “putting their lives on the line” when there were no life insurance policies for them. One of the participants had reported that hazard allowance for health workers was five thousand naira per month (US$13.2/month: Central bank of Nigeria exchange rate). Although, the hazard allowance of health workers in Lagos state which had the highest number of cases of COVID-19 during the study period witnessed an increase from US$13 to US$65 to incentivize them [11], there is still need for an overhaul of the current policy towards improving health workers moral for efficient service delivery during pandemics. Participants in this study revealed that stigmatization of patients by health workers was an important factor which discouraged pregnant women from seeking MNCH services. COVID-19 was regarded at as a death sentence” during the initial period of the pandemic and as such health workers discriminated against anybody who had symptoms similar to COVID-19. This was compounded by the lack of knowledge regarding COVID-19 and inadequacy of PPEs which made critical assessment difficult. Although our study did not evaluate the effect of these barriers during the lockdown on maternal and perinatal outcomes, a study from Zimbabwe reported a decrease in utilization of maternal health services and an increased risk of adverse maternal and neonatal outcomes during the nationwide lockdown [29].

The highlighted challenges in this study faced by service users and providers in accessing and delivering MNCH services respectively during the COVID-19 pandemic closely resembles the problems identified during Ebola virus outbreaks in three West African countries [8]. This suggests that disease outbreaks and pandemics impacts on MNCH services in similar patterns. Therefore, concerted efforts need to be made to address the identified problems towards sustaining MNCH services during pandemics.

Our study showed that similar barriers to MNCH services existed across all the three levels of health care delivery system in Nigeria but was more in the high burdened states of Lagos, Abuja and Kano compared to the low burdened states of Bayelsa, Taraba and Enugu. This may have been due to the high cases of COVID-19 in the former states which would imply increase compliance to lockdown measures, increased negative socio-economic impact, heightened fear of contracting COVID-19 by both patients and health workers as well as higher tendency for nonavailability of personal protective equipment in those states.

Previous studies conducted during the pre-COVID-19 period had reported poor attitude of health workers, poor health seeking behaviours, long waiting times and high cost of health care services as major reasons for non-utilization of MNCH services in Nigeria [30, 31]. Our study which was carried out during the first wave of COVID-19 corroborated these findings but also highlighted that these existing problems were further worsened by the lockdowns. Although the problem of lack of transportation and distance of service users from the hospital had existed before the COVID-19 pandemic, the element of harassment of patients by law enforcement agents in the bid to implement the lockdown was a new perspective of barriers women could face in accessing MNCH services in Nigeria. Additionally, patients expressed fear presenting to the hospital due to their perceived increased risk of getting infected with COVID-19 virus at the hospitals.

Despite the barriers, our study identified facilitators of access to and delivery of MNCH services during the first six months of COVID-19 pandemic in Nigeria. These facilitators should be further improved upon towards increased service delivery after the COVID-19 pandemics. These include the need for continued community education regarding the disease, modification of health care consultation practices, training, and supportive supervision of health workers regarding safe practices during disease outbreaks and adaptation of available guidelines and protocols for uninterrupted service delivery. These measures were much more operational in the primary health care centers across the six states due to the early community sensitization and training of health workers by the National Primary Health Care Development Agency.

The strength of the study was the involvement of three stakeholder groups (service users, health care workers and policy makers). This allowed for examination of the subject matter from various perspectives. Additionally, participants were drawn from the six geopolitical zones of the country, three geopolitical zones of the states and across the three levels of Nigeria’s public health service system. This provided a nationally representative group for generalizability of the study findings.

This study was however limited by the fact that the interviews was conducted using the phone which limited assessment of nonverbal cues which are important aspects of qualitative studies. Furthermore, this may result in selection bias due to the inclusion of only service-users of higher socioeconomic status in the study. However, mobile phone interviews have an advantage of respondents being relaxed and willing to talk freely thereby allowing for collection of detailed high-quality data [32]. The use of mobile phones for data collection in this study became necessary due to the need for interviewer and respondents’ safety as well as the lockdowns which limited travels.

It is envisaged that the scientific evidence generated from this study will be used for the development of policies and interventions that will assist Nigeria in maintaining focus towards improving maternal and newborn child health amidst the COVID-19 pandemic. This can be achieved by addressing underlying reasons for non-utilization of MNCH services during infectious disease outbreaks and ensure prioritization of sustenance of MNCH services by early community sensitization to allay fears, provision of social safety nets to cushion economic difficulties, special transportation arrangements and incentives for health workers, training of health workers and provision of PPEs for patients and health workers safety.

## Conclusion

Access to MNCH services were negatively affected by lockdown during the first wave of COVID-19 pandemic in Nigeria particularly due to challenges resulting from restrictions in movements which affected patients/healthcare providers ability to reach the hospitals as well as patients’ ability to pay for health care services. Additionally, there was fear of contracting COVID-19 infection at health facilities and the health systems inability to provide enabling conditions for sustained utilization of MNCH services. There is need for government to institute alternative measures to halt the spread of diseases instead of lockdowns so as to ensure unhindered access to MNCH services during future pandemics. This may include immediate sensitization of the general public on modes of transmission of any emergent infectious disease as well as training of health workers on emergency preparedness and alternative service delivery models.

## Supplementary Information

**Additional file 1.**

## Data Availability

The data sets generated during and/or analyzed during the current study are available from the corresponding author on reasonable request.

## Abbreviations

IDIs: In-depth interviews
MNCH: Maternal, newborn and child health
PPE: Personal protective equipment
SP: Service Providers
SU: Service Users
PM: Policy Makers
