# Supplementary material for: Barriers and facilitators of access to maternal, newborn and child health services during the first wave of COVID-19 pandemic in Nigeria: findings from a qualitative study
Source: BMC Health Serv Res. 2022 May 6;22:611. doi: 10.1186/s12913-022-07996-2 (PMC9073814; doi:10.1186/s12913-022-07996-2)
Supplement: Supplementary file 1 — Additional file 1. [file 12913_2022_7996_MOESM1_ESM.docx]

**In-depth Interview (IDI) Guide for Service Users (Women for antenatal, delivery, postnatal, immunization, family planning services)**

Please use this guide to facilitate in-depth interviews with service users. This guide will elicit questions on service users’ experiences in accessing MNCH services in the ongoing COVID-19 pandemic.

Participants should be thoroughly probed for information. Researchers to start by introducing themselves and a brief about the project. They should collect the information needed in the table below before proceeding with the questions.

**BACKGROUND INFORMATION**

| In-depth interview code: |  |
| --- | --- |
| In-depth Interview date: |  |
| Narrative Interviewer’s name: |  |
| Interview start time |  |
| Interview end time |  |
| Religion of participant: |  |
| Gender of the participant: |  |
| Age of participant: |  |
| Age of Child (if nursing) |  |
| Name of Health Facility |  |
| State in which interview was conducted |  |
| Language in which narrative interview was undertaken: |  |
| Informed Consent given by participant (Purpose of the interview and confidentiality explained. Informed Consent forms signed and collected: |  |
| Verbal Consent given by participant to audio record the narrative interview: |  |

**In-depth Interview Guide**

| **S/N** | **Questions** | **Probes** |
| --- | --- | --- |
| 1. | When was your first visit to this clinic (pre or during pandemic)  Where and how did you get your first information about COVID-19?  Kindly explain what you heard about COVID-19 pandemic? | Medium of information  Person responsible for the information  Transmission route, symptoms  Safety/preventive measures  PPE obtainable |
| 2 | Have you had any health education session for COVID-19?  If yes, kindly explain in details and state how helpful it was. | Where and When? |
| 3 | What are the MNCH services that are readily accessible in this facility in this period of COVID-19 pandemic? | ANC, labour and delivery, routine immunization, childcare, FP etc. |
| 4 | Kindly mention the MNCH services that you accessed during this period of COVID-19 pandemic. (State reasons)  If ANC services (Please verify if registration took place during COVID-19). If yes, how easy, or difficult was it?  Were there home deliveries? (You or other women in your community you know and why they discontinued usage of health facilities?)  If FP services, any Change of reproductive plans during Covid-19? (state reasons) | ANC, routine immunization, childcare, FP etc.  Movement restrictions  Financial reasons  Unavailability of FP methods in health facilities (for instance women who already have intrauterine devices may find it difficult to get them changed as some clinics may have paused such service) etc. |
| 5  6  7 | Kindly mention the MNCH services that you couldn’t access during this period of COVID-19 pandemic.  What are the challenges/barriers you experienced accessing MNCH services during this period?  How can these challenges/barriers be mitigated? | Delay in seeking care factors (Lack of information on availability of services during the COVID-19 pandemic, finance etc).  Delay in reaching care (availability of transportation services, distance to health facilities etc.)  Delay in receiving adequate healthcare at the hospital. |
| 8 | Have you observed any change as regards accessing MNCH services this period? If yes, what are they? (State reasons for such observed changes) | Standard of healthcare provision  Medical commodities  Patronage  Staff strength  Staff attitude  Working hours  Diverted attention to Covid-19 related cases |
| 9 | How safe do you think it is for women to access MNCH services in this facility this period? | Perceived competence of Service providers  Precautions taken  Safety measures employed  Use of PPEs  Hygiene practice |
| 10  11 | Were you anxious about anything this period and how did that affect you?  Kindly explain any kind of support received while accessing MNCH services in the ongoing pandemic | Fertility and family planning, giving birth, childcare, from school closure, care in families as most domestic staff were not available, etc.  Support from family members, spouse, healthcare workers, government etc. |

## **In-depth Interview (IDI) Guide for Service Providers**

Please use this guide to facilitate in-depth interviews with service providers. This guide will elicit questions on service providers experiences in providing MNCH services in the ongoing COVID-19 pandemic.

Participants should be thoroughly probed for information. Researchers to start by introducing themselves and a brief about the project. They should collect the information needed in the table below before proceeding with the questions.

**BACKGROUND INFORMATION**

| In-depth interview code: |  |
| --- | --- |
| In-depth Interview date: |  |
| Narrative Interviewer’s name: |  |
| Designation of Participant: |  |
| Interview start time |  |
| Interview end time |  |
| Religion of participant: |  |
| Gender of the participant: |  |
| Age of participant: |  |
| Name of Health Facility |  |
| State in which interview was conducted |  |
| Language in which narrative interview was undertaken: |  |
| Informed Consent given by participant (Purpose of the interview and confidentiality explained. Informed Consent forms signed and collected: |  |
| Verbal Consent given by participant to audio record the narrative interview: |  |

**Interview Guide**

| **S/N** | **Questions** | **Probes** |
| --- | --- | --- |
| 1 | Where and how did you get your first information about COVID-19?  Kindly explain what you heard about COVID-19 pandemic? | Medium of information  Person responsible for the information  Transmission route  Safety/preventive measures  PPE obtainable |
| 2 | In your opinion, what do you consider as essential health services?  Which of these essential services do you provide in this health facility? | MNCH services  Emergency services etc. |
| 3 | What are the MNCH services readily available in this facility in this period of COVID-19 pandemic | ANC, labour and delivery, immunization, routine immunization, childcare, FP etc. |
| 4 | Kindly list the MNCH services that is being accessed by clients this period. (State reasons)  Were service users reluctant to access MNCH services this period?  Were there home deliveries (reasons) | ANC, labour and delivery, post-natal care, routine immunization, FP etc.  Were there any MNCH services that were on hold this period?  Which services were predominantly affected? (Give reasons)  Effects of the lockdown on MNCH services? |
| 5 | Kindly list the MNCH services that are not being accessed by clients this period? (State reasons)  Were there any deaths this period associated with lack of access to MNCH services? | Delay in seeking care (socio-economic status, religious and cultural beliefs, poor information about COVID-19, influence of family members)  Delay in reaching care (availability of transportation services due to movement restriction, distance to health facilities etc.)  Delay in receiving adequate healthcare (opening hours, lack of PPEs, inadequate medical commodities, shortage of manpower) |
| 7 | Were there MNCH outreach programmes carried out this period? (Kindly list them)  If no, were there alternative strategies for immunization activities or other MNCH outreaches? | Immunization  Awareness/sensitization programme |
| 8 | Has there been any observed change as regards provision of MNCH services this period? If yes, what are they? (State reasons for such observed changes) | Standard of healthcare provision  Medical commodities  Patronage  Staff strength  Working hours |
| 9 | What is the service regimen for MNCH service delivery during this period of the pandemic? | Precautions taken  Safety measures employed  Use of PPEs  Hygiene practice  Availability of SOPs  Quality assurance checklist/tools  Waste disposal protocol |
| 10 | Were there referrals this period? | If yes, kindly describe how it was handled and if no, give reasons. |
| 11 | How do you feel as a health care provider during this period of the ongoing pandemic?  Kindly explain some of the coping/adaptive mechanisms that has helped you while providing services. | Juggling caring for patients and taking care of families (particularly with closure of schools, unavailability of domestic staff, etc.)  Perceptions regarding contacting COVID-19 at workplace etc. |
| 12 | What are the challenges/barriers MNCH care providers in this facility has experienced in providing services during this period? (How can they be mitigated) | Lockdown factors, funding for health,  Inadequate/unavailability of PPEs, medical commodities etc.  Availability of human resources |

## **In-depth Interview (IDI) Guide For Policy Makers**

Please use this guide to facilitate in-depth interviews with policy makers. This guide will elicit questions on policy makers knowledge about the policies, strategies and action plans associated with MNCH services in the ongoing COVID-19 pandemic.

Participants should be thoroughly probed for information. Researchers to start by introducing themselves and a brief about the project. They should collect the information needed in the table below before proceeding with the questions.

**BACKGROUND INFORMATION**

| In-depth interview code: |  |
| --- | --- |
| In-depth Interview date: |  |
| Narrative Interviewer’s name: |  |
| Interview start time |  |
| Interview end time |  |
| Religion of participant: |  |
| Gender of the participant: |  |
| Age of participant: |  |
| Name of Health Facility |  |
| State in which interview was conducted |  |
| Language in which narrative interview was undertaken: |  |
| Informed Consent given by participant (Purpose of the interview and confidentiality explained. Informed Consent forms signed and collected: |  |
| Verbal Consent given by participant to audio record the narrative interview: |  |

**Interview Guide**

| S/N | Questions | Probes |
| --- | --- | --- |
|  | Kindly explain what you know about COVID-19 pandemic? | Transmission route  Safety/preventive measures  PPE obtainable |
|  | What measures have been put in place by the government, ministry of health or hospital management as regards MNCH services since the pandemic started? | Safety measures (Provision of PPEs)  Information sharing  Funding  Availability of medical commodities  Provision of transportation services for health workers |
|  | Are there key policies, strategies and action plans for MNCH services since the pandemic started? (kindly state them) | If yes, what is the implementation status?  If no, what are the plans to develop one? |
|  | What are the quality assurance checklists or tools for ensuring quality delivery of MNCH services during this pandemic? | Are there existing standard operational guidelines?  Has the institution adapted any International guideline/recommendations? |
|  | What the challenges/barriers that are being experienced in delivery of MNCH services during this period of COVID-19? | How can these challenges/barriers be mitigated? |
